# Association between waiting time and survival in patients undergoing local treatment for biliary tract cancer: a nationwide study

**DOI:** 10.1007/s00423-026-04170-8

**Published:** 2026-08-11

**Authors:** Diana Elena Renteria Ramirez, Lucas Alexander Knøfler, Jakob Kirkegård, Alice Markussen, Claus Wilki Fristrup, Mogens Tornby Stender, Delal Akdag, Susanne Dam Nielsen, Hasan Ahmad Al-Saffar, Hans-Christian Pommergaard

**Affiliations:** 1 Hepatic Malignancy Surgical Research Unit (HEPSURU), Department of Digestive Diseases, Transplantation and General Surgery, Copenhagen University Hospital, Rigshospitalet, Blegdamsvej 9, Copenhagen, 2100 Denmark; 2 Department of Surgery, Aarhus University Hospital, Aarhus, Denmark; 3 Department of Oncology, Copenhagen University Hospital, Herlev and Gentofte, Herlev , Denmark; 4 Department of Surgery, Odense University Hospital, Odense, Denmark; 5 Department of Surgery, Aalborg University Hospital, Aalborg, Denmark; 6 Institute for Clinical Medicine, University of Copenhagen, Panum Institute, Copenhagen, Denmark; 7 Department of Infectious Diseases, Copenhagen University Hospital, Rigshospitalet, Copenhagen, Denmark

**Keywords:** Biliary tract cancer, Wait to treatment, Survival, Local treatment

## Abstract

**Purpose:**

Biliary tract cancers are aggressive malignancies with low survival rates. Therefore, the delays in receiving treatment could represent a challenge to successful outcomes. However, recent evidence suggests that extended waiting times for surgery in digestive cancers do not affect survival. This study investigated the association between the interval from multidisciplinary team (MDT)-based suspected diagnosis to initiation of local treatment and survival among patients with biliary tract cancer.

**Methods:**

This was a nationwide, multicenter, cohort study based on data from patients locally treated for intrahepatic cholangiocarcinoma, perihilar cholangiocarcinoma, and gallbladder cancer from 2013 to 2023.

**Results:**

Registry data from 303 patients were analyzed. The median waiting time from diagnosis to treatment for the entire cohort was 28.0 days, with an interquartile range of 21.0–41.0 days. Multivariable restricted cubic spline analysis suggested a statistically significant and a borderline non-linear, association between waiting time to treatment and overall survival (overall Wald χ2 = 67.5, *p* = < 0.0001; non-linear Wald χ2 = 5.8, *p* = 0.053; C-index = 0.33). Doubtful operability and resection of extrahepatic bile ducts were associated with long waits in intrahepatic cholangiocarcinoma (p = < 0.001 and *p* = 0.003, respectively). Futile laparotomy was associated with short waiting times in gallbladder cancer (*p* = 0.027).

**Conclusion:**

We observed a potential U-shaped, non-linear relationship between waiting time and survival. Kaplan-Meier analyses did not identify statistically significant differences according to the median waiting time. Doubtful operability, resection of extrahepatic bile ducts, and futile laparotomy were associated with waiting time to local treatment.

**Supplementary Information:**

The online version contains supplementary material available at https://doi.org/10.1007/s00423-026-04170-8.

## Introduction

 Cholangiocarcinoma and gallbladder cancer are distinct malignancies within the group of biliary tract cancers. Biliary tract cancers are characterized by an increasing incidence and low overall survival rates of less than 20% at five years, primarily due to late-stage detection [1]. Both cholangiocarcinoma and gallbladder cancer have unique risk factors and must be individually considered due to their unique morphology and treatment approaches [1, 2]. Surgery is the only curative option and is feasible for approximately 25% and 10% of cholangiocarcinoma and gallbladder cancer cases, respectively [3, 4]. For patients with unresectable or metastatic disease who are not candidates for curative resection, alternative local therapies such as stereotactic radiotherapy and transarterial radioembolization may be considered [5].

In certain European countries, legal regulations dictate the time between cancer diagnosis and treatment initiation [6]. Cumulative evidence has shown that every four-week delay in receiving surgery may represent a 6–8% increase in the risk of mortality for cancers of the bladder, breast, colon, rectum, lung, cervix, and head and neck [7]. However, controversy has arisen as different groups of authors from Europe and the United States have concluded that extended waiting times for surgery in digestive cancers was not associated with increased mortality [8–12]. The primary aim of this study was to assess the influence of waiting time from suspected diagnosis at Multidisciplinary Team (MDT) conference to treatment on survival in a cohort of patients with biliary tract cancer who underwent local treatment as the primary treatment within a national framework with applied legislation on maximum waiting time. As a secondary aim, we examined the association between clinical characteristics and final operative result and waiting time.

## Materials and methods

### Study design, sources, and participants

This was a nationwide, multicenter, cohort study based on data from patients diagnosed with perihilar cholangiocarcinoma, intrahepatic cholangiocarcinoma, and gallbladder cancer from 2013 to 2023, identified in the Danish Liver Cancer Group registry, prospectively maintained by the Danish Healthcare Quality Institute. Distal cholangiocarcinoma was not included in this study since they are not captured by the database. All patients were diagnosed, referred, and surgically treated at Danish hospitals after an MDT conference following the standard course times specified in section 88 of the Danish Health Act. According to this regulation, both medical and surgical cancer treatments must be initiated within 28 calendar days from the date the hospital receives the referral for treatment [13].

**Fig. 1:**
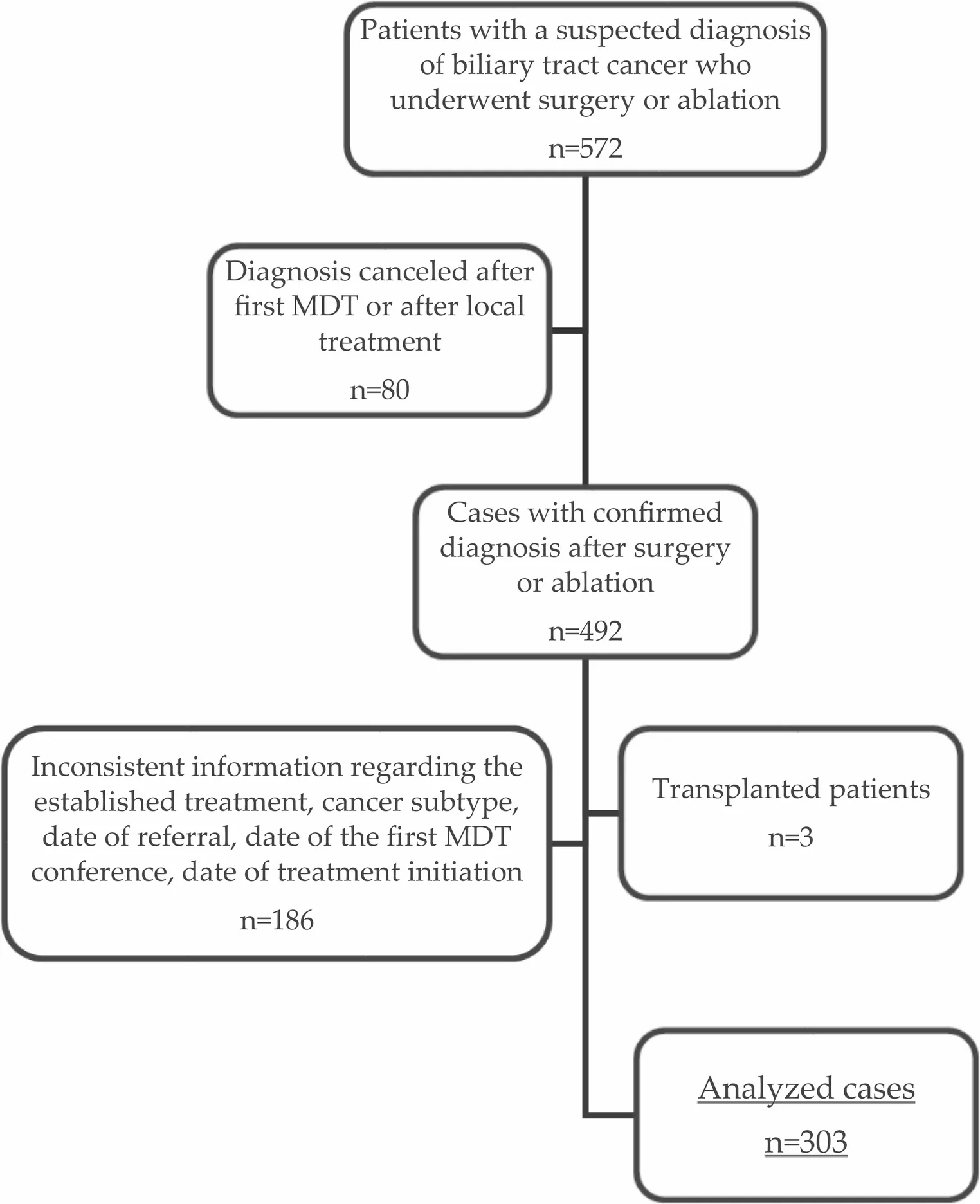
Flow diagram of the study population

Patients with the following characteristics were excluded: (1) those who received cancer therapy other than local treatment as first treatment, (2) patients with diagnosis of biliary tract cancer waived during the MDT conference or after treatment, (3) transplant recipients or patients who underwent portal vein embolization, (4) patients with inconsistent or unspecified information regarding the treatment method, the date of referral, date of first/last MDT conference, and date of treatment (e.g., inconclusive MDT course at the time of treatment or date of treatment more than 100 days after the first MDT conference) (Fig. 1).

### Outcome variables

Survival was defined as the time from the date of treatment to either the date of death retrieved from the Danish Civil Registration number [14], regardless of cause, or the last follow up date (15/11/2023). The database has subsequently been terminated. Waiting time was defined as the number of calendar days between the date of referral to the first MDT conference (considered initial diagnosis) and the date of the first treatment procedure. This interval included the time from referral to the first MDT conference as well as the time from MDT assessment to treatment initiation. The workup period was calculated as the number of days between the date of the first MDT and the last MDT conference of the ongoing course.

### Statistical analysis

Descriptive characteristics were presented as medians with interquartile range (IQR) for numerical data, and as frequencies and percentages for categorical data.

For handling missing data (≤ 40%), a random forest method was conducted using the missForest package version 1.5. The model was based on the following variables: age, sex, Eastern Cooperative Oncology Group performance status score (ECOG-PS), biliary cancer subtype, operability, resectability (based on the assessment by the referring department), days of workup, waiting time from diagnosis to treatment, local treatment, tumor stage, time of survival, and event (death).

Waiting time from diagnosis to local treatment was analyzed for the entire cohort and by biliary tract cancer subtype; data are presented as median with IQR. Survival analysis was conducted using the Kaplan-Meier estimator, presented with a 95% confidence interval (95% CI). Patients were categorized according to whether their values were above or below the median and compared using the log-rank test. Waiting time to local treatment was compared across treatment groups using the Kruskal-Wallis test after assessing the distribution of the data. Pairwise comparisons were performed with Bonferroni adjustment.

Restricted cubic splines (RCS) were used to model the time-varying and non-linear association between waiting time to treatment and overall survival, with waiting time modeled as a continuous variable. The multivariable RCS model was adjusted for covariates including age, sex, ECOG-PS, tumor stage, operability, resectability, local treatment, and workup. Both RCS models were performed using the rms package (version 8.0–0). Four knots were placed at the 5th, 35th, 65th, and 95th percentiles. The median waiting time was used as the reference value. The proportional hazards assumption was evaluated using Schoenfeld residuals. The overall significance of the spline term and evidence of non-linearity were tested using Wald χ². Model performance was evaluated using the concordance index (C-index).

Univariable and multivariable linear regression models within cancer subgroups were employed to investigate the association between clinically relevant variables and waiting time from diagnosis to treatment. Variables with cases in less than 2 categories were excluded from the multivariable analysis. Model fit was evaluated using the R-squared statistic. Regression coefficients (β), 95% CI, and p-values were reported for each variable. Analyses were conducted in RStudio version 2024.04.2.

### Regulatory approvals

The Danish Data Protection Agency approved the study (p-2023-14848). Ethical approval and informed consent were not required for this study according to the Danish law.

The manuscript adheres to the reporting guidelines outlined in the STROBE Statement [15].

## Results

### Patient cohort

A total of 303 patients were included in the analysis (Fig. 1). Table 1 and Supplementary Table 1 summarize the descriptive characteristics of the cohort and of each cancer subtype, respectively. In 61.0% of the analyzed cases, the workup required to establish the treatment plan was completed in a single session of the MDT conference.

**Table 1:** Descriptive characteristics of the cohort

Age	
Years, median (IQR)	69.6 (61.4–75.0)
Sex	
Female	173 (57.1)
Male	130 (42.9)
Cancer subtype	
Intrahepatic cholangiocarcinoma	115 (38.0)
Perihilar cholangiocarcinoma	64 (21.1)
Gallbladder cancer	124 (40.9)
ECOG-PS	
0–1	213 (70.3)
2	20 (6.6)
3–4	1 (0.3)
Missing	69 (22.8)
Operability	
Yes	290 (95.7)
No	2 (0.7)
Doubtful	11 (3.6)
Resectability	
Yes	302 (99.7)
No	1 (0.3)
Workup	
Days, median (IQR)	0.0 (0.0–14.0)
Tumor stage	
Stage 1 or 2	162 (53.5)
Stage 3 or 4	51 (16.8)
Missing	90 (29.7)
Operative results	
Liver resection	224 (73.9)
Resection of extrahepatic bile ducts	57 (18.8)
Futile laparotomy	18 (5.9)
Ablation	4 (1.3)

*IQR* Interquartile range,* MDT* multidisciplinary team,* ECOG-PS* Eastern Cooperative Oncology Group performance status score

**Table 2:** Univariable and multivariable analyses of factors associated with waiting time from diagnosis to local treatment in surgical patients with biliary tract cancer

A. Intrahepatic cholangiocarcinoma						
	**Univariable**			**Multivariable**		
	**Coefficient (β)**	**95% CI**	**p-value**	**Coefficient (β)**	**95% CI**	**p-value**
Sex						
Female	Ref.	-	-	Ref.	-	-
Male	-1.07	-5.21, 7.36	0.735	0.64	-5.23, 6.53	0.876
Age						
Years	0.16	-0.16, 0.49	0.325	0.20	-0.11, 0.51	0.207
ECOG-PS						
0 - 1	Ref.	-	-	Ref.	-	-
2	-2.40	-14.75, -9.95	0.701	-8.40	-20.82, 4.00	0.182
3 - 4	NA	NA	NA	NA	NA	NA
Operability						
Yes	Ref.	-	-	Ref.	-	-
No	7.34	-24.86, 39.55	0.652	5.74	-25.30, 36.79	0.714
Doubtful	34.00	13.58, 53.38	**<0.001**	33.56	14.48, 52.64	**<0.001**
Tumor stage						
Stage 1 or 2	Ref.	-	**-**	Ref.	-	**-**
Stage 3 or 4	-3.52	-11.01, 3.97	0.354	-4.28	-12.31, 3.75	0.293
Operative result						
Liver resection	Ref.	-	-	Ref.	-	-
Ablation	2.34	-13.85, 18.54	0.774	6.30	-10.47, 23.08	0.457
Resection of extrahepatic bile ducts	19.96	8.28, 31.65	**<0.001**	17.52	5.92, 29.12	**0.003**
Futile laparotomy	-12.62	-25.04, -0.19	**0.046**	-7.47	-21.56, 6.61	0.295
B. Perihilar cholangiocarcinoma						
	**Univariable**			**Multivariable**		
	**Coefficient (β)**	**95% CI**	**p-value**	**Coefficient (β)**	**95% CI**	**p-value**
Sex						
Female	Ref.	-	-	Ref.	-	-
Male	-5.55	-13.81, 2.70	0.184	-5.09	-13.62, 3.44	0.237
Age						
Years	0.24	-0.12, 0.61	0.192	0.24	-0.13, 0.63	0.205
ECOG-PS						
0 - 1	Ref.	-	-	Ref.	-	-
2	NA	NA	NA	NA	NA	NA
3 - 4	15.87	-17.41, 49.15	0.344	13.40	-20.71, 47.52	0.434
Operability						
Yes	Ref.	-	-	-	-	-
No	-13.58	-46.93, 19.76	0.419	-16.69	-51.04, 17.66	0.335
Doubtful	NA	NA	NA	NA	NA	NA
Tumor stage						
Stage 1 or 2	Ref.	-	-	Ref.	-	-
Stage 3 or 4	-7.92	-16.00, 0.16	0.054	-6.49	-15.40, 2.41	0.150
Operative result						
Liver resection	Ref.	-	-	Ref.	-	-
Ablation	NA	NA	NA	NA	NA	NA
Resection of extrahepatic bile ducts	4.47	-4.49, 13.44	0.322	4.35	-4.69, 13.40	0.339
Futile laparotomy	2.19	-18.34, 22.72	0.832	2.31	-18.64, 23.27	0.826
C. Gallbladder cancer						
	**Univariable**			**Multivariable**		
	**Coefficient (β)**	**95% CI**	**p-value**	**Coefficient (β)**	**95% CI**	**p-value**
Sex						
Female	Ref.	-	-	Ref.	-	-
Male	-1.95	-7.66, 3.76	0.500	-2.49	-8.37, 3.37	0.401
Age						
Years	-0.27	-0.27, 0.23	0.871	-0.06	-0.32, 0.19	0.608
ECOG-PS						
0 - 1	Ref.	-	-	Ref.	-	-
2	-0.25	-9.18, 8.67	0.955	3.21	-9.60, 16.03	0.620
3 - 4	NA	NA	NA	NA	NA	NA
Operability						
Yes	Ref.	-	-	-	-	-
No	NA	NA	NA	NA	NA	NA
Doubtful	-0.20	-10.94, 10.54	0.970	4.38	-10.95, 19.72	0.572
Tumor resectability						
Yes	NA	NA	NA	NA	NA	NA
No	-2.08	-31.59, 27.43	0.889	10.78	-21.88, 43.44	0.514
Operative result						
Liver resection	Ref.	-	-	Ref.	-	-
Ablation	NA	NA	NA	NA	NA	NA
Resection of extrahepatic bile ducts	-6.26	-16.31, 3.79	0.220	-5.43	-15.77, 4.89	0.229
Futile laparotomy	-9.77	-20.39, 0.84	0.071	-15.66	-29.52, -1.79	**0.027**

*Adjusted R-squared = 0.16*

*Adjusted R-squared= 0.10*

*Adjusted R-squared= 0.001*

### Waiting time and survival analyses

**Fig. 2:**
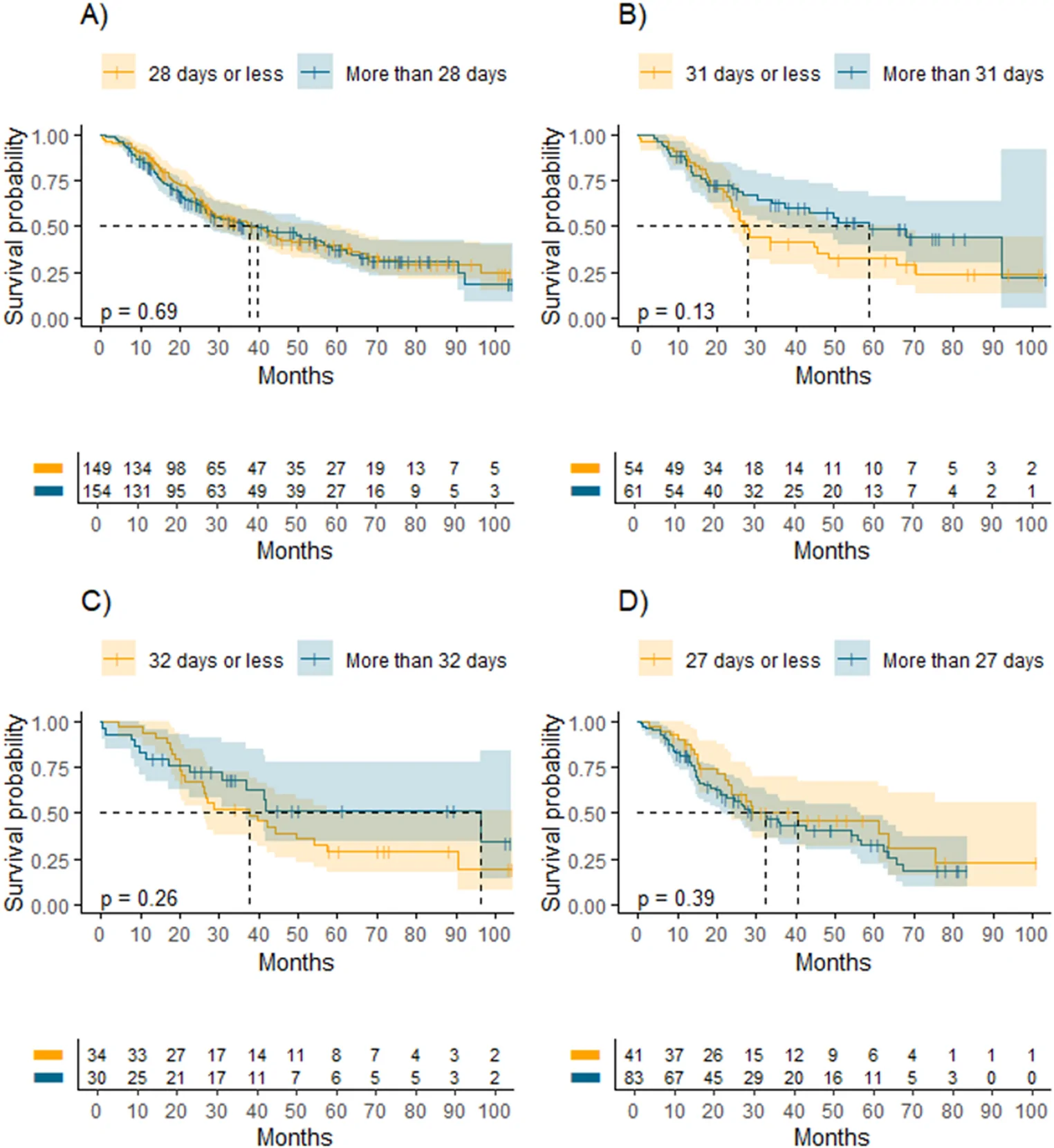
Kaplan-Meier estimators for survival (months) according waiting time from diagnosis to local treatment in different cancer groups: (**A**) Biliary tract cancer, (**B**) Intrahepatic cholangiocarcinoma, (**C**) Perihilar cholangiocarcinoma, and (**D**) Gallbladder cancer

The median survival for intrahepatic cholangiocarcinoma was 43.6 months (95%CI 27.6–68.0), 41.7 months (95%CI 28.5–96.1) for perihilar cholangiocarcinoma, and 32.4 months (95%CI 24.4–56.5) for gallbladder cancer, respectively. Overall survival did not differ significantly between the groups in the comparative analysis (*p* = 0.25). For the entire cohort, the median waiting time from diagnosis to treatment was 28.0 days (IQR 21.0–41.0) and survival was similar between patients classified as below or above 28 days (*p* = 0.69) (Fig. 2A). When organized by cancer subtype, the median waiting time from diagnosis to treatment for patients with intrahepatic cholangiocarcinoma was 31.0 days (IQR 20.0-43.5), for those with perihilar cholangiocarcinoma was 32.0 days (IQR 21.0-42.2), and 27.0 days (IQR 20.0–35.0) for patients with gallbladder cancer. There was a trend toward a difference in survival between cancer subtypes, but this did not reach statistical significance (*p* = 0.061). Kaplan-Meier curves for each cancer subtype did not show a statistically significant difference in overall survival rates between the patients above and below the median of waiting time from diagnosis to local treatment (Fig. 2B-D). The waiting time to treatment was statistically similar across operative result groups and is reported in Supplementary Table 2.

**Fig. 3:**
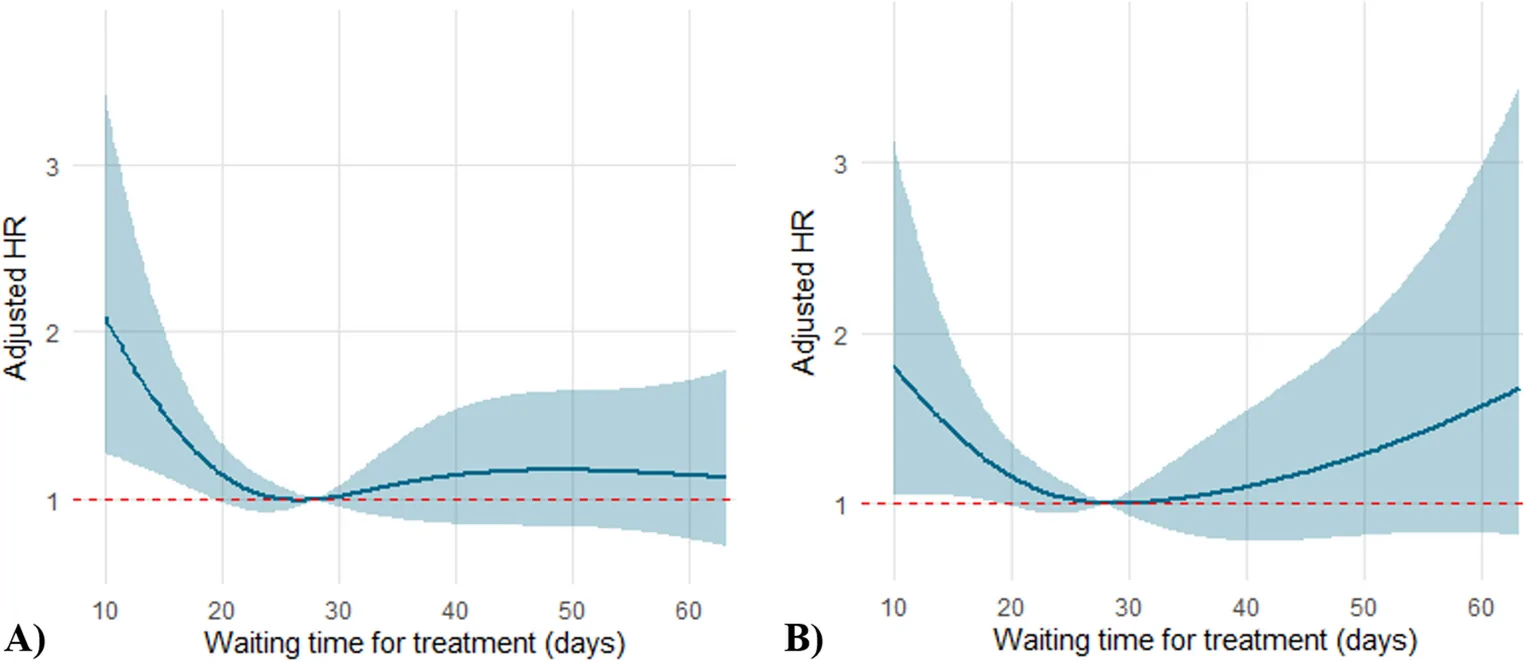
Association between waiting time to treatment and overall survival with the restricted cubic splines function in the entire cohort. (**A**) Univariable model, (**B**) Multivariable model

The univariable RCS analysis suggested a statistically significant and non-linear association between waiting time to treatment and overall survival (overall Wald χ² = 8.84, *p* = 0.031; non-linear Wald χ²= 6.93, *p* = 0.031; C-index = 0.45) (Fig. 3A). The HR for overall survival was 1.72 (95% CI 1.19–2.48) at the 5th percentile (day 13), 1.03 (95% CI 0.92–1.15) at the 35th percentile (day 23), 1.07 (95% CI 0.89–1.29) at the 65th percentile (day 34), and 1.14 (95% CI 0.75–1.73) at the 95th percentile (day 61) (Fig. 3A). In the multivariable RCS analysis, the overall association with survival was statistically significant, while non-linearity was borderline (overall Wald χ² = 67.5, *p* = < 0.0001; non-linear Wald χ² = 5.8, *p* = 0.053; C-index = 0.33) (Fig. 3B). Similar to the univariable model, decreased survival was observed with both longer and shorter waiting time in relation to the median.

**Fig. 4:**
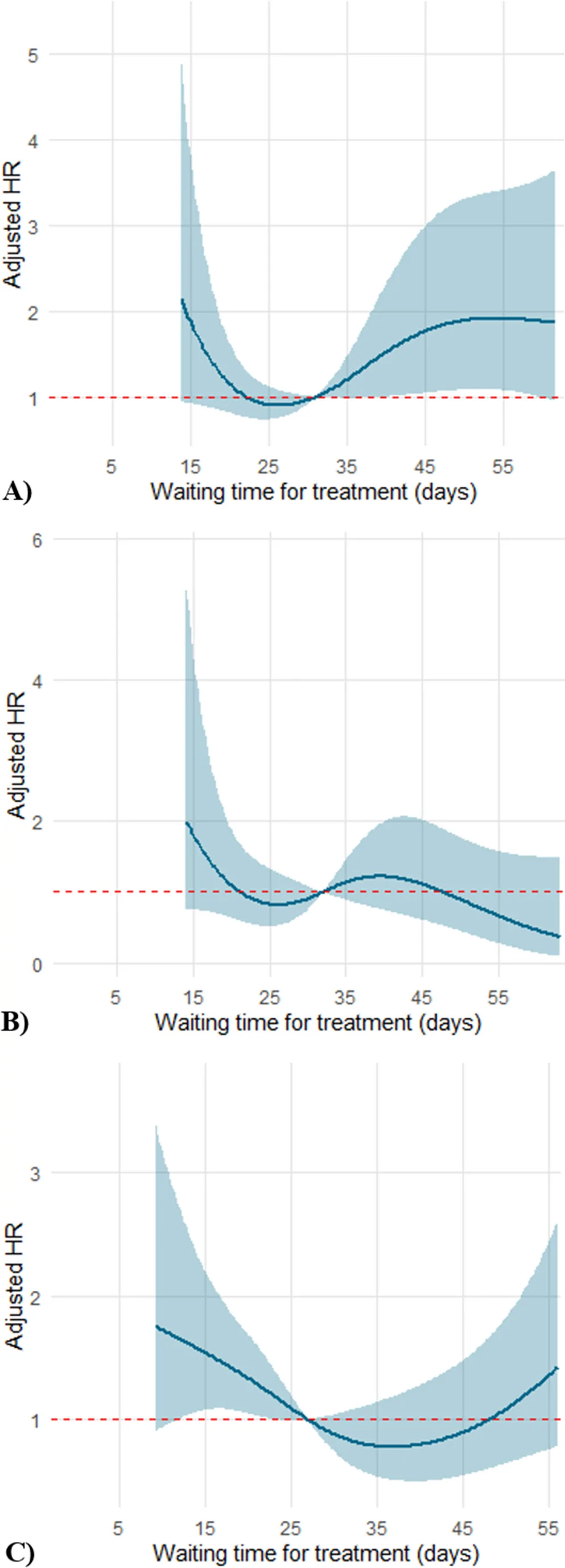
Association between waiting time to treatment and overall survival analyzed using the univariable restricted cubic splines function in: (**A**) Intrahepatic cholangiocarcinoma, (**B**) Perihilar cholangiocarcinoma, and (**C**) Gallbladder cancer

Subsequently, RCS analyses were then performed for each cancer subgroup to examine whether the overall association, mainly driven by shorter waiting times, was explained by a specific subgroup. A borderline significant overall association with non-linear pattern was found in gallbladder cancer (overall Wald χ² = 7.77, *p* = 0.051; non-linear Wald χ² = 7.71, *p* = 0.021; C-index = 0.42) (Fig. 4C). For intrahepatic (overall Wald χ² = 5,52, *p* = 0.137; non-linear Wald χ² = 5.04, *p* = 0.080; C-index = 0.42) and perihilar cholangiocarcinoma (overall Wald χ² = 5,15, *p* = 0.161; non-linear Wald χ² = 2.54, *p* = 0.280; C-index = 0.39) no associations were observed (Fig. 4A and B). In all cases, the model showed poor discrimination, suggesting no clear evidence that waiting time influences survival in these subgroups.

### Patient’s characteristics and operative results associated with waiting time

For intrahepatic cholangiocarcinoma, univariable linear regression analysis demonstrated that doubtful operability (compared to operability classified as “yes”) and resection of extrahepatic bile ducts were independently associated with longer waiting time to treatment, whereas futile laparotomy was associated with shorter waiting time (compared with liver resection). In this cancer subgroup, the multivariable linear regression analysis confirmed the associations only in doubtful operability (β = 33.56 [95% CI 14.48, 52.64], p = < 0.001) and resection of extrahepatic bile ducts (β = 17.52 [95% CI -5.92, 29.12], *p* = 0.003) (Table 2A). In gallbladder cancer, univariable linear regression showed a trend toward significance for the association between futile laparotomy and waiting time (*p* = 0.071), with liver resection as the reference; significance was reached after adjusting for covariates (β= -15.66 [95% CI -29.52, -1.79], *p* = 0.027) (Table 2C). No significant associations were found in the perihilar subgroup (Table 2B).

## Discussion

While some authors support a connection between long waiting time and poor survival [7, 16–18], others reject this association [12, 19, 20]. To address this controversy, the present study investigated the association of waiting time to local treatment in a multicenter national cohort of patients diagnosed with biliary tract cancer. Our results demonstrated no differences in survival when comparing groups with waiting times below versus above the median. RCS analyses revealed a non-linear association between waiting time and survival, with poorer outcomes observed both in patients treated shortly after the first MDT conference and in those who experienced long delays before treatment. Our analysis also showed that doubtful operability at the time of diagnosis and resection of the extrahepatic bile ducts as the initial treatment were associated with longer waiting times, whereas futile laparotomy was associated with shorter waiting times.

In contrast with previous studies conducted in patients with biliary tract cancer [12, 21], we found an association between waiting time to receive treatment and survival. However, the multivariable RCS analysis of gallbladder cancer suggested a possible U-shaped association between waiting time and overall survival. Specific prognostic patient features associated with both long and short waiting times may explain this, suggesting that waiting time could be a potential confounder in the association between disease stage and survival. If a causal relationship existed, we would have expected a gradually increasing impact on prognosis with longer waiting times. The higher risk of mortality in patients with longer waiting times could be explained by the fact that more extensive preoperative workup is usually required for more advanced disease. Although patients pre-treated with portal vein embolization were excluded from this study, other preoperative interventions, such as biliary drainage and liver function tests (e.g., indocyanine green [ICG] clearance test), which are used for more complex surgeries, typically prolong waiting time. The association between worse prognosis and shorter waiting times may be a consequence of patients experiencing futile laparotomy due to under staged disease, resulting in non-curative treatment. It is hypothesized that these patients may have been under staged because of insufficient preoperative workup, allowing a shorter interval to surgery. However, this hypothesis could not be confirmed with the available data.

The median waiting time from diagnosis to treatment in the entire cohort was 28 days. After the individual analysis of each cancer subtype, statistics showed that survival was not affected by exceeding waiting time, in respect to the median. Yet, from a clinical perspective, notable differences in survival were observed for intrahepatic and perihilar cholangiocarcinoma cases with waiting times above the corresponding median that cannot rule out a type-2 error. Previous studies have also reported the waiting times to surgery for intrahepatic [12] and perihilar cholangiocarcinoma individually [21]. For intrahepatic cholangiocarcinoma, authors categorized the waiting time from the date of diagnosis to the date of surgery in 30 days intervals, reporting no significative difference in survival [12]. In cases with perihilar cholangiocarcinoma, it has been reported a median waiting time of 64 days in surgical patients, concluding that receiving surgery before or after the median did not affect survival [21]. Although both conclusions are consistent with our results, the heterogeneity in study designs made comparisons difficult. Notably, to our knowledge, this is the first multicenter study conducted with this aim; therefore, our findings provide more generalizable data for this population.

Gallbladder cancer is usually detected incidentally (in up to 70% of cases) after surgery initially performed for gallstone disease. Based on the T-stage in the specimen, particularly for patients with confirmed T1b, T2, and T3 tumors, re-resection is recommended to minimize the risk of residual disease [22, 23]. The optimal period to conduct the re-resection is not clear, however, a period of 4 to 8 weeks after the initial cholecystectomy has been related with better survival (23). In our cohort, patients with gallbladder cancer had a median waiting time of 27 days. Conversely, a Dutch study reported a median waiting time of 75 days from the primary surgery and the re-resection operation (24). Unfortunately, due to the nature of the database, our results do not distinguish whether the surgery in patients with gallbladder cancer was a primary surgery conducted under a confirmed diagnosis of malignancy or a re-resection after elective cholecystectomy for suspected benign disease.

Associations between patient’s characteristics and operative results and waiting time were identified in our analysis, and among the studied covariates, doubtful operability was associated with longer waiting times in patients with intrahepatic cholangiocarcinoma. Careful preoperative evaluation is of particular importance in hepatobiliary surgery, given the increased surgical risk observed in elderly patients. However, it is now accepted that elderly patients with low comorbidities and good overall health can achieve outcomes after hepatobiliary surgery for oncologic disease comparable to those of their younger counterparts [24]. Preoperative optimization can lead in longer waiting time for treatment, as procedures such as portal vein embolization or biliary drainage might be needed. The impact of preoperative optimization was evaluated in a European tertiary-center cohort, showing that although treatment delays may occur, prolonged workup did not influence survival [25]. Moreover, a recent randomized clinical trial in patients with sarcopenia scheduled for major liver resection and requiring future liver remnant hypertrophy showed a marked reduction in overall morbidity, from 50% to 13.3%, among participants in a 6-week prehabilitation program. This indicates that prehabilitation may actually be beneficial for patients before surgery, even with the extended waiting times for treatment [26].

The present study, moreover, provides evidence on how waiting time was influenced by the therapeutic procedure. Longer waits for treatment were found in patients with intrahepatic cholangiocarcinoma treated with resection of extrahepatic bile ducts, likely due to the complexity of surgical planning. On the other hand, shorter waiting times were observed before futile laparotomy. This intervention was performed in less than 6% of the cohort, whereas other studies reported futile laparotomy in one third of surgical patients [27]. We suggest that this lower rate may reflect better preoperative patient selection by the MDT and improved imaging diagnostics, which reduce discrepancies between pre- and intraoperative findings.

Among the limitations of this registry-based study is the presence of missing data, particularly for the variables ECOG-PS and tumor stage. This was a database analysis spanning 10 years and involving four hospitals, then documentation errors are possible. Our database did not include registries of distal cholangiocarcinoma, which limited the study of this subgroup. Patients with planned surgery or other local treatments who ultimately did not undergo the procedure, as well as those who received portal vein embolization—a special group with distinct timelines—were excluded from this study. We acknowledge the potential for selection bias in our material, since tumor progression during the waiting period may have led to deferral of treatment. In the case of portal vein embolization, longer waits are expected due to the time required for growth of the future liver remnant. However, the database did not provide sufficient information to properly analyze or adjust for this bias. In addition, we excluded patients whose initial treatment took place more than 100 days after the first MDT conference, as in our clinical practice this reflects a realistic threshold beyond which updated imaging is typically required to reassess disease progression. Additionally, the lack of preoperative biochemical parameters and comorbidity data further limited the scope of our analyses. Therefore, we encourage future studies to include additional parameters with demonstrated clinical relevance.

## Conclusions

No association between waiting time and survival was found when comparing patients with waiting times below and above the median. However, the multivariable RCS analysis of gallbladder cancer suggested a U-shaped pattern, whereby both short and long waiting times, relative to the median, appeared to be associated with a higher hazard of death. Thus, while this association is not considered causal, it likely reflects confounding, where extreme deviations from the median waiting time indicate an abnormal disease course associated with worse prognosis.

## Supplementary Information

Below is the link to the electronic supplementary material.

Supplementary Material 1

## Data Availability

No datasets were generated or analysed during the current study.

## References

[1] Lamarca A, Edeline J, Goyal L (2022) How I treat biliary tract cancer. ESMO Open 7. 10.1016/j.esmoop.2021.100378 PMC8762076 35032765

[2] Vogel A, Bridgewater J, Edeline J, Kelley RK, Klümpen HJ, Malka D et al (2023) Biliary tract cancer: ESMO Clinical Practice Guideline for diagnosis, treatment and follow-up. Ann Oncol 34:127–140. 10.1016/j.annonc.2022.10.506 36372281

[3] Banales JM, Marin JJG, Lamarca A, Rodrigues PM, Khan SA, Roberts LR et al (2020) Cholangiocarcinoma 2020: the next horizon in mechanisms and management. Nat Rev Gastroenterol Hepatol 17:557–588. 10.1038/s41575-020-0310-z PMC7447603 32606456

[4] Sturm N, Schuhbaur JS, Hüttner F, Perkhofer L, Ettrich TJ (2022) Gallbladder Cancer: Current Multimodality Treatment Concepts and Future Directions. Cancers (Basel) 14:5580. 10.3390/cancers14225580 PMC9688543 36428670

[5] Owen M, Makary MS, Beal EW (2023) Locoregional Therapy for Intrahepatic Cholangiocarcinoma. Cancers (Basel) 15. 10.3390/cancers15082384 PMC10137284 37190311

[6] Osowiecka K, Rucinska M, Nowakowski JJ, Nawrocki S (2018) How long are cancer patients waiting for oncological therapy in Poland? Int J Environ Res Public Health 15. 10.3390/ijerph15040577 PMC5923619 29570661

[7] Hanna TP, King WD, Thibodeau S, Jalink M, Paulin GA, Harvey-Jones E et al (2020) Mortality due to cancer treatment delay: systematic review and meta-analysis. BMJ 371:m4087. 10.1136/bmj.m4087 PMC7610021 33148535

[8] Xu K, Watanabe-Galloway S, Rochling FA, Farazi PA, Monirul Islam KM, Wang H et al (2019) Surgical Delay Is Associated with Improved Survival in Hepatocellular Carcinoma: Results of the National Cancer Database. J Gastrointest Surg 23:933–943. 10.1007/s11605-018-3925-4 PMC12110860 30328070

[9] Kirkegård J, Mortensen FV, Hansen CP, Mortensen MB, Sall M, Fristrup C (2019) Waiting time to surgery and pancreatic cancer survival: A nationwide population-based cohort study. Eur J Surg Oncol 45:1901–1905. 10.1016/j.ejso.2019.05.029 31160135

[10] Mirkin KA, Hollenbeak CS, Wong J (2018) Time to Surgery: a Misguided Quality Metric in Early Stage Pancreatic Cancer. J Gastrointest Surg 22:1365–1375. 10.1007/s11605-018-3730-0 29520648

[11] Jooste V, Dejardin O, Bouvier V, Arveux P, Maynadie M, Launoy G et al (2016) Pancreatic cancer: Wait times from presentation to treatment and survival in a population-based study. Int J Cancer 139:1073–1080. 10.1002/ijc.30166 27130333

[12] Mantas A, Liu D, Otto CC, Heij LR, Heise D, Bruners P et al (2024) Time to surgery is not an oncological risk factor in patients with cholangiocarcinoma undergoing curative-intent liver surgery. Sci Rep 14. 10.1038/s41598-023-50842-6 PMC10796920 38238432

[13] Bekendtgørelse om maksimale ventetider ved behandling af kræft og visse tilstande (2025) ved iskæmiske hjertesygdomme n.d. https://www.retsinformation.dk/eli/lta/2015/584. Accessed 8 Sept

[14] Pedersen CB (2011) The Danish Civil Registration System. Scand J Public Health 39:22–25. 10.1177/1403494810387965 21775345

[15] von Elm E, Altman DG, Egger M, Pocock SJ, Gøtzsche PC, Vandenbroucke JP (2008) The Strengthening the Reporting of Observational Studies in Epidemiology (STROBE) statement: guidelines for reporting observational studies. J Clin Epidemiol 61:344–349. 10.1016/j.jclinepi.2007.11.008 18313558

[16] Elit L (2015) Wait times from diagnosis to treatment in cancer. J Gynecol Oncol 26:246–248. 10.3802/jgo.2015.26.4.246 PMC4620358 26404128

[17] Cone EB, Marchese M, Paciotti M, Nguyen DD, Nabi J, Cole AP et al (2020) Assessment of Time-to-Treatment Initiation and Survival in a Cohort of Patients With Common Cancers. JAMA Netw Open 3:E2030072. 10.1001/jamanetworkopen.2020.30072 PMC7737088 33315115

[18] Khorana AA, Tullio K, Elson P, Pennell NA, Grobmyer SR, Kalady MF et al (2019) Time to initial cancer treatment in the United States and association with survival over time: An observational study. PLoS One 14. 10.1371/journal.pone.0213209 PMC6396925 30822350

[19] Hangaard Hansen C, Gögenur M, Tvilling Madsen M, Gögenur I (2018) The effect of time from diagnosis to surgery on oncological outcomes in patients undergoing surgery for colon cancer: A systematic review. Eur J Surg Oncol 44:1479–1485. 10.1016/j.ejso.2018.06.015 30251641

[20] Castelo M, Paszat L, Hansen BE, Scheer AS, Faught N, Nguyen L et al (2023) Analysis of Time to Treatment and Survival among Adults Younger Than 50 Years of Age with Colorectal Cancer in Canada. JAMA Netw Open 6:E2327109. 10.1001/jamanetworkopen.2023.27109 PMC10401304 37535356

[21] Tohmatsu Y, Ohgi K, Ashida R, Yamada M, Otsuka S, Kato Y et al (2024) Time to Surgery Does Not Affect the Survival Outcome in Patients with Perihilar Cholangiocarcinoma. Ann Surg Oncol. 10.1245/s10434-024-16628-4 39633168

[22] Ethun CG, Postlewait LM, Le N, Pawlik TM, Buettner S, Poultsides G et al (2017) Association of Optimal Time Interval to Re-resection for Incidental Gallbladder Cancer With Overall Survival. JAMA Surg 152:143. 10.1001/jamasurg.2016.3642 PMC5800764 27784058

[23] van Dooren M, de Savornin Lohman EAJ, Brekelmans E, Vissers PAJ, Erdmann JI, Braat AE et al (2023) The diagnostic value of staging laparoscopy in gallbladder cancer: a nationwide cohort study. World J Surg Oncol 21. 10.1186/s12957-022-02880-z PMC9840315 36641472

[24] Papis D, Vagliasindi A, Maida P (2020) Hepatobiliary and pancreatic surgery in the elderly: Current status. Ann Hepatobiliary Pancreat Surg 24:1. 10.14701/ahbps.2020.24.1.1 PMC7061045 32181422

[25] Ruys AT, Heuts SG, Rauws EA, Busch ORC, Gouma DJ, Van Gulik TM (2014) Delay in surgical treatment of patients with hilar cholangiocarcinoma: Does time impact outcomes? HPB 16:469–474. 10.1111/hpb.12156 PMC4008165 24033549

[26] Berardi G, Cucchetti A, Colasanti M, Angrisani M, Moschetta G, Chiappori D et al (2025) Prehabilitation With Exercise and Nutrition to Reduce Morbidity of Major Hepatectomy in Patients With Sarcopenia. JAMA Surg. 10.1001/jamasurg.2025.3102 PMC12392143 40864449

[27] Goel M, Varty GP, Patkar S, Meghana V, Kazi M, Nandy K et al (2024) Preventing futile surgery in Intrahepatic and Perihilar cholangiocarcinomas: Can we identify preoperative factors to improve patient selection and optimize outcomes? Surg Oncol 55:102096. 10.1016/j.suronc.2024.102096 38964224

