# Supplementary material for: Association between waiting time and survival in patients undergoing local treatment for biliary tract cancer: a nationwide study
Source: Langenbecks Arch Surg. 2026 Aug 11;411(1):309. doi: 10.1007/s00423-026-04170-8 (PMC13743587; doi:10.1007/s00423-026-04170-8)
Supplement: Supplementary file 1 [file 423_2026_4170_MOESM1_ESM.docx]

**Supplementary material**

**Supplementary Table 1. Clinical characteristics and treatment details by biliary cancer subtype.**

|  | Intrahepatic cholangiocarcinoma (n=115) | Perihilar cholangiocarcinoma (n=64) | Gallbladder cancer  (n=124) |
| --- | --- | --- | --- |
| Age | | | |
| Median (IQR) | 69.8 (63.5-75.3) | 67.6 (60.8-73.8) | 70.2 (61.4-75.1) |
| Sex | | | |
| Female | 59 (51.3) | 28 (43.8) | 86 (68.4) |
| Male | 56 (48.7) | 36 (56.2) | 38 (30.6) |
| ECOG-PS | | | |
| 0 – 1 | 80 (69.6) | 58 (90.6) | 75 (60.5) |
| 2 | 8 (7.0) | 0 (0.0) | 12 (9.7) |
| 3 -4 | 0 (0.0) | 1 (1.6) | 0 (0.0) |
| Missing | 27 (23.4) | 5 (7.8) | 37 (29.8) |
| Operability | | | |
| Yes | 111 (96.5) | 63 (98.4) | 116 (93.5) |
| No | 1 (0.9) | 1 (1.6) | 0 (0.0) |
| Doubtful | 3 (2.6) | 0 (0.0) | 8 (6.5) |
| Resectability | | | |
| Yes | 115 (100.0) | 64 (100.0) | 123 (99.2) |
| No | 0 (0.0) | 0 (0.0) | 1 (0.8) |
| Workup | | | |
| Median (IQR) | 0.0 (0.0-16.0) | 14.0 (0.0-26.2) | 0.0 (0.0-0.0) |
| Tumor stage ^a, b^ | | | |
| Stage 1 or 2 | 83 (72.2) | 31 (48.4) | 48 (38.7) |
| Stage 3 or 4 | 23 (20.0) | 28 (43.8) | 0 (0.0) |
| Missing | 9 (7.8) | 5 (7.8) | 76 (61.3) |
| Operative result | | | |
| Liver resection | 96 (83.5) | 21 (32.8) | 107 (86.3) |
| Resection of extrahepatic bile ducts | 8 (7.0) | 40 (62.5) | 9 (7.3) |
| Futile laparotomy | 7 (6.1) | 3 (4.7) | 8 (6.4) |
| Ablation | 4 (3.4) | 0 (0.0) | 0 (0.0) |

*IQR,* *Interauartile range; MDT, multidisciplinary team; ECOG-PS,* *Eastern Cooperative Oncology Group performance status score.*

**Supplementary Table 2. Waiting time (days) organized by local treatment in the entire cohort.**

| **Operative result** | **Median (IQR)*** | **Patients treated before day 28, (n, %**)** | **Patients treated after day 28, (n, %**)** |
| --- | --- | --- | --- |
| Liver resection | 28.0 (20.0-41.0) | 116 (75.3) | 108 (72.5) |
| Resection of extrahepatic bile ducts | 33.0 (21.0-48.0) | 25 (16.2) | 32 (21.5) |
| Futile laparotomy | 23.0 (11.8-30.8) | 12 (7.8) | 6 (4.0) |
| Ablation | 34.0 (27.0-42.2) | 1 (0.7) | 3(2.0) |

**Global test p=0.055; pairwise comparisons analysis p= >0.05 across all groups.*

***Proportion of the group.*
